# A cystic lesion adjacent to the cystic duct diagnosed as ectopic thyroid tissue: a laparoscopic case report

**DOI:** 10.3389/fsurg.2026.1751389

**Published:** 2026-06-26

**Authors:** Jingnan Xue, Xiaohai Wu, Longhao Zhang, Jiangqin Liu, Xiaoli He, Nanzi Xie, Xin Lu, Gang Xie, Kai Zhou

**Affiliations:** 1 Department of Hepatobiliary Surgery, People’s Hospital of Anshun City, Guizhou, China; 2 Department of Liver Surgery, Peking Union Medical College Hospital, Chinese Academy of Medical Sciences & Peking Union Medical College, Beijing, China; 3 Department of Thyroid and Breast Surgery, People’s Hospital of Anshun City, Guizhou, China; 4 Department of Pathology, People’s Hospital of Anshun City, Guizhou, China

**Keywords:** ectopic thyroid tissue, gallbladder neck, hilar mass, laparoscopic, orthotopic thyroid

## Abstract

Ectopic thyroid tissue is an uncommon developmental anomaly, and involvement of the hepatobiliary region—particularly near the gallbladder neck or cystic duct—is extremely rare. Such lesions may mimic primary biliary or gallbladder masses, creating diagnostic and therapeutic uncertainty. We report a 50-year-old woman with a 2-year history of intermittent right upper quadrant abdominal pain. Contrast-enhanced CT demonstrated a well-defined hyperdense lesion at the hepatic hilum adjacent to the gallbladder neck, measuring 22 × 14 mm (∼80 HU on non-contrast; 119 HU arterial; 104 HU venous), with indistinct borders from the gallbladder. A biliary or gallbladder-associated tumor could not be excluded, and further evaluation with MRI and PET-CT was recommended. The patient declined additional imaging due to financial constraints and strongly requested operative management. Laparoscopic cholecystectomy with *en bloc* resection of the hilar lesion was performed. Intraoperative frozen-section analysis and final histopathology revealed benign ectopic thyroid tissue composed of mature, well-differentiated thyroid follicles without atypia. Postoperative thyroid ultrasound showed a normal orthotopic thyroid gland. The patient recovered uneventfully and remained symptom-free at follow-up. Ectopic thyroid tissue adjacent to the gallbladder neck is an exceptionally rare cause of a hilar mass. Surgeons should consider this entity in the differential diagnosis of gallbladder neck or porta hepatis lesions of uncertain origin and rely on histopathology to guide appropriate, conservative management.

## Introduction

Ectopic thyroid tissue is defined as thyroid tissue located outside its normal pretracheal position, resulting from abnormal migration of the thyroid anlage during embryogenesis (1). The most common site is along the thyroglossal duct tract, particularly at the base of the tongue, while subdiaphragmatic locations are distinctly uncommon (2). Ectopic thyroid has been sporadically reported in the mediastinum, lung, heart, liver, porta hepatis, gallbladder, gastrointestinal tract, pancreas, and ovary (3–5).

Ectopic thyroid tissue involving the gallbladder or structures in close proximity to the gallbladder neck is exceedingly rare. Case reports have described ectopic thyroid tissue within the gallbladder wall, in duplicated gallbladder, and in the gallbladder bed, often discovered incidentally at or after cholecystectomy. More recently, several reports have documented ectopic thyroid in the porta hepatis or hepatoduodenal ligament, sometimes mimicking primary hepatobiliary or lymph node pathology and necessitating surgical resection for definitive diagnosis.

Preoperative diagnosis of abdominal ectopic thyroid is challenging, as clinical manifestations are usually nonspecific and cross-sectional imaging findings overlap with those of other benign and malignant lesions (6). Fine-needle aspiration or core needle biopsy may not always be feasible due to anatomical constraints or patient preference, leading to diagnostic uncertainty and a tendency toward surgical exploration.

We present a rare case of ectopic thyroid tissue located adjacent to the gallbladder neck and hepatic hilum, initially suspected to be a biliary or gallbladder-related mass. The lesion was managed laparoscopically and diagnosed intraoperatively as ectopic thyroid on frozen-section analysis. This case highlights important considerations in preoperative assessment, intraoperative decision-making, and postoperative evaluation, and is discussed in the context of the existing literature on hepatobiliary and abdominal ectopic thyroid.

## Case description

A 50-year-old woman presented with a 2-year history of intermittent right upper quadrant abdominal pain. The pain was dull, non-radiating, and unrelated to meals. She denied fever, chills, jaundice, nausea, vomiting, weight loss, or symptoms suggestive of thyroid dysfunction, such as palpitations, heat or cold intolerance, or neck swelling. Her medical and surgical history were unremarkable, and she was not taking any regular medications.

On physical examination, she was afebrile and hemodynamically stable. There was mild tenderness in the right upper quadrant without guarding, rebound, or palpable mass. No cervical lymphadenopathy or thyroid enlargement was noted. Routine laboratory tests, including complete blood count, liver function tests, serum bilirubin, amylase, and inflammatory markers, were within normal limits. Postoperative thyroid function tests were also normal (Table 1).

**Table 1:** Postoperative thyroid function.

Date	T3(noml/L)	T4(noml/L)	FT3(poml/L)	FT4(poml/L)	TSH(ulU/mL)
2025-04-06	2.37	115.00	5.82	16.80	4.340
2025-08-03	2.45	131.00	4.73	14.24	4.220
2026-01-28	2.33	125.00	4.86	15.22	3.860

Contrast-enhanced abdominal CT revealed a well-defined patch-like hyperdense lesion in the hepatic hilum adjacent to the gallbladder neck. The lesion measured approximately 22 × 14 mm, with a mean attenuation of about 80 Hounsfield units (HU) on non-contrast images. Following intravenous contrast administration, the lesion demonstrated moderate enhancement, with attenuation values of 119 HU in the arterial phase and 104 HU in the venous phase (Figure 1). The boundary between the lesion and the gallbladder neck was indistinct, raising concern for a gallbladder neck–related mass, hilar lymph node enlargement, or other biliary tract lesion of uncertain origin.

**Figure 1:**
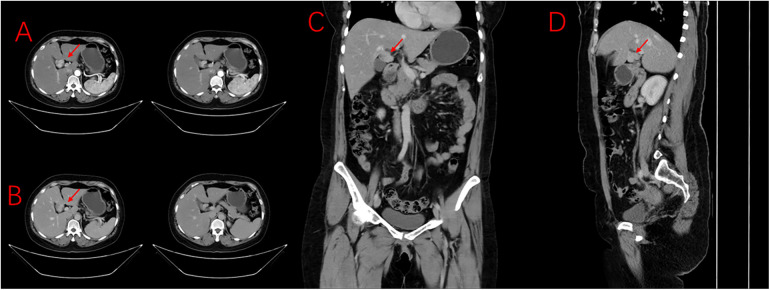
Preoperative contrast-enhanced CT of the abdomen. **(A,B)** Axial arterial and portal venous phase images show a well-defined, mildly hyperdense lesion at the hepatic hilum adjacent to the gallbladder neck (red arrows). The interface between the lesion and the gallbladder/cystic duct is indistinct. **(C,D)** Coronal reconstructed and sagittal reconstructed image demonstrates the lesion located beside the gallbladder neck and porta hepatis, without obvious invasion of the surrounding liver parenchyma or major vessels.

Given the indeterminate nature of the lesion, contrast-enhanced MRI and PET-CT were recommended to further characterize the mass and exclude malignancy. However, due to financial constraints, the patient and her family declined additional imaging and strongly requested definitive surgical treatment after being informed of the potential risks and benefits.

Laparoscopic exploration was performed. Intraoperatively, the gallbladder showed chronic cholecystitis changes. A cystic lesion was identified adjacent to the cystic duct and gallbladder neck, extending toward the hepatic hilum, without obvious invasion of the liver parenchyma, common bile duct, or surrounding vascular structures (Figure 2). Laparoscopic cholecystectomy combined with *en bloc* excision of the para–cystic duct hilar lesion was carried out. The specimen was sent for intraoperative frozen-section analysis.

**Figure 2:**
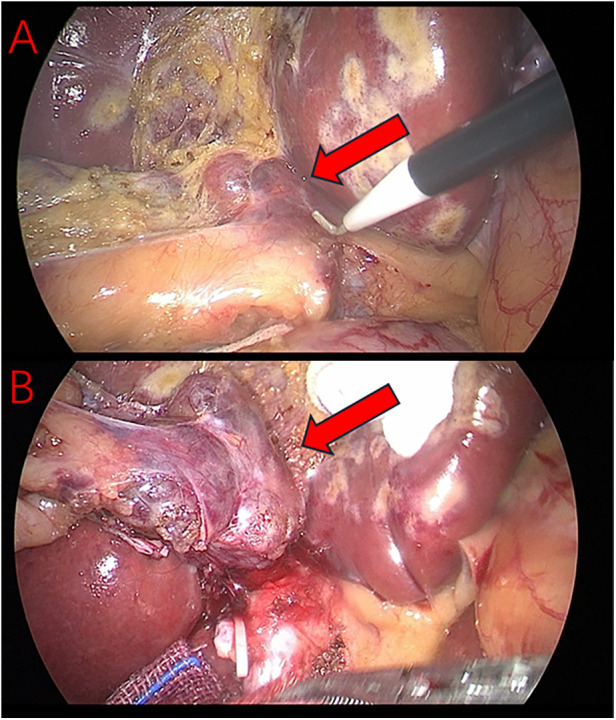
Intraoperative laparoscopic views. **(A)** Initial laparoscopic exploration reveals a cystic mass (red arrow) located beside the cystic duct and gallbladder neck, extending toward the hepatic hilum. **(B)** After careful dissection, the para–cystic duct lesion (red arrow) is further exposed and prepared for *en bloc* resection together with the gallbladder.

Frozen-section examination revealed thyroid-like tissue composed of well-formed follicles filled with colloid, consistent with ectopic thyroid tissue and without features of malignancy. Based on this result, no further radical resection was undertaken. Final histopathology confirmed benign ectopic thyroid tissue, composed of mature follicular structures lined by uniform follicular epithelial cells lacking nuclear atypia, papillary structures, capsular invasion, or vascular invasion. supporting the diagnosis of ectopic thyroid (Figure 3).

**Figure 3:**
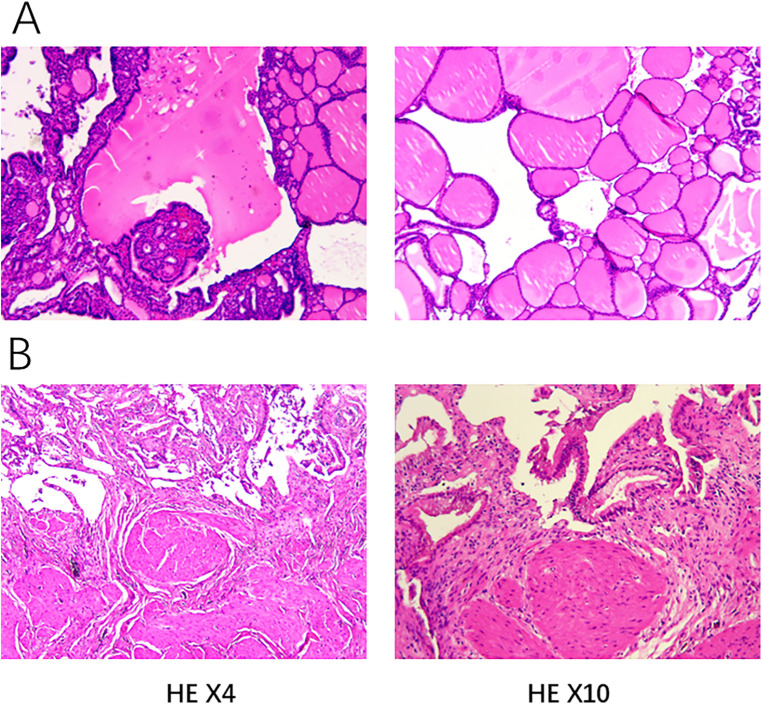
Histopathological features of the lesion (H&E staining). **(A)** Low-power (×4), intermediate-power (×10) views show ectopic thyroid tissue composed of well-formed thyroid follicles filled with eosinophilic colloid. The follicles are lined by uniform cuboidal follicular cells without nuclear atypia, papillary structures, capsular invasion or vascular invasion, consistent with benign ectopic thyroid tissue. **(B)** The gallbladder shows diffuse infiltration of chronic inflammatory cells, predominantly lymphocytes and plasma cells.

The postoperative course was uneventful. The patient resumed oral intake on postoperative day 1 and was discharged on postoperative day 3 in good condition. A postoperative neck ultrasound revealed a normally located thyroid gland without nodules or suspicious lesions. At clinical follow-up (e.g., 6–12 months), she remained asymptomatic with no radiological evidence of recurrence.

### Diagnostic assessment

The main diagnostic challenge in this case was the characterization of an incidentally detected hilar lesion adjacent to the gallbladder neck. The patient's symptoms and laboratory tests were nonspecific. Contrast-enhanced CT demonstrated a small, moderately enhancing lesion with an indistinct interface with the gallbladder neck, which raised suspicion for:

- Gallbladder neck mass (e.g., adenomyoma, adenoma, early carcinoma)
- Enlarged hilar lymph node
- Other benign or malignant biliary tract lesion

Because of the lesion's location and enhancement pattern, malignancy could not be confidently excluded. Additional imaging with contrast-enhanced MRI and PET-CT was advised to further delineate tissue characteristics, assess metabolic activity, and evaluate for distant disease. However, these studies were declined for economic reasons. Preoperative biopsy was not pursued due to the small size and deep hilar location of the lesion, with concern for bleeding and bile duct injury.

Thus, the working diagnosis remained an indeterminate hilar mass adjacent to the gallbladder neck, and surgery was planned both for symptom relief (chronic cholecystitis) and diagnostic/therapeutic management of the lesion.

### Therapeutic intervention

The patient underwent laparoscopic cholecystectomy with *en bloc* resection of the para–cystic duct hilar lesion. Standard four-port laparoscopy was used. The cystic duct and artery were carefully dissected, and the lesion adjacent to the gallbladder neck and hepatic hilum was identified as a cystic mass without obvious invasion of neighboring structures. After securing the cystic duct and artery, the lesion was excised with surrounding soft tissue to achieve complete removal and avoid bile duct injury.

Intraoperative frozen-section analysis of the mass revealed benign ectopic thyroid tissue. In the absence of malignant features, no additional extended hepatic or biliary resection or lymphadenectomy was performed.

### Follow-up and outcomes

Postoperatively, the patient received routine analgesia and prophylactic antibiotics according to institutional practice. She recovered smoothly, with no bile leak, hemorrhage, or other complications. Oral intake and mobilization were resumed on postoperative day 1, and she was discharged on day 3.

Postoperative neck ultrasound confirmed a normal orthotopic thyroid gland without nodules. Thyroid function remained normal. At follow-up (e.g., 6–12 months after surgery), the patient reported complete resolution of right upper quadrant pain, and there was no clinical or imaging evidence of recurrence (Box 1).

BOX 1 **Timeline.**

**Table:** 

Time point	Event and findings
2023-07	Onset of intermittent right upper quadrant pain
2025-04-01	Hospital admission; physical exam and labs normal
2025-04-03	Contrast-enhanced CT: 22 × 14 mm hilar lesion adjacent to gallbladder neck, indeterminate nature
2025-04-03	Recommendation for MRI and PET-CT; patient declined due to financial constraints
2025-04-03	Laparoscopic cholecystectomy + resection of para–cystic duct hilar lesion
2025-04-03	Frozen section: benign ectopic thyroid tissue
2025-04-06	Discharged uneventfully
2025-04-08	Thyroid ultrasound: normal orthotopic thyroid, no nodules
2025-08-03	Asymptomatic; no evidence of recurrence

## Discussion

This case illustrates an exceptionally rare presentation of ectopic thyroid tissue located adjacent to the gallbladder neck and hepatic hilum, initially mimicking a biliary or gallbladder neck mass. Most ectopic thyroid tissue is found along the midline cervical route of thyroid migration, particularly at the base of the tongue and infrahyoid region; subdiaphragmatic locations are uncommon. Ectopic thyroid involving the gallbladder or hepatobiliary structures has been reported only sporadically, including cases within the gallbladder wall, duplicated gallbladder, gallbladder bed, porta hepatis, and hepatoduodenal ligament (7, 8).

Our case shares several features with previously reported hepatobiliary ectopic thyroid cases. Patients are often middle-aged women presenting with nonspecific abdominal pain or are completely asymptomatic. Imaging typically reveals a well-circumscribed lesion whose enhancement pattern overlaps with that of other benign and malignant entities. In this context, preoperative diagnosis of ectopic thyroid is rarely achieved, and definitive identification usually relies on histopathology, with or without supportive immunohistochemistry.

Complete excision is considered curative for benign disease, and thyroid hormone replacement is typically unnecessary because most patients retain a normally located, functioning thyroid gland. We propose a risk-proportionate follow-up strategy: a postoperative clinical review with baseline thyroid function testing (TSH and FT4, T4, FT3, T3) at 1–3 months, repeat testing at 6–12 months, and annual monitoring thereafter if stable. Additional imaging of the operative region (ultrasound/CT/MRI) is not routinely required and should be reserved for new biliary symptoms or incidental imaging abnormalities.

A key clinical concern is distinguishing benign ectopic thyroid tissue from metastatic thyroid carcinoma, particularly in patients with a history of thyroid malignancy or nodular thyroid disease. In our patient, there was no history of thyroid disease, thyroid function was normal, and postoperative ultrasound demonstrated a normal orthotopic thyroid gland. Histologically, the lesion consisted of mature thyroid follicles without atypia, papillary nuclear features, or invasive growth, supporting a benign ectopic thyroid rather than metastatic disease.

From a surgical standpoint, this case underscores the importance of careful intraoperative assessment and the value of frozen-section analysis. In the absence of a preoperative diagnosis and given the lesion's hilar location, a more aggressive resection (such as extended hepatic or bile duct resection) might have been considered if malignancy were suspected. Intraoperative confirmation of benign ectopic thyroid tissue allowed the surgical team to avoid unnecessary radical surgery and its associated morbidity.

The strengths of our approach include thorough preoperative counseling, minimally invasive management with laparoscopic surgery, and use of intraoperative frozen section to guide the extent of resection. However, several limitations should be acknowledged. First, advanced imaging (MRI, PET-CT) was not performed due to financial constraints, which might have provided additional diagnostic clues. Second, immunohistochemistry was not strictly necessary given the classic morphology, but it can be useful in challenging cases to confirm thyroid origin and exclude other entities.

The take-away lessons from this case are:

1. Ectopic thyroid tissue, although rare, should be considered in the differential diagnosis of small, indeterminate lesions near the gallbladder neck, porta hepatis, or hepatoduodenal ligament.
2. Preoperative imaging alone may not permit a definitive diagnosis; histopathology remains the gold standard.
3. Intraoperative frozen-section examination is particularly valuable to prevent overtreatment when malignancy is uncertain.
4. Postoperative evaluation of the orthotopic thyroid is important to exclude synchronous primary thyroid disease or metastasis.

## Patient perspective

**“**The pain in my right upper abdomen had been bothering me for a long time, and I was worried it might be something serious. When the doctors told me there was a mass near my gallbladder and that more tests were recommended, I was anxious but could not afford additional expensive examinations. I agreed to have the surgery because I wanted a clear answer and relief from the pain. After the operation, I was relieved to learn that the mass was benign ectopic thyroid tissue. My recovery was smooth, my pain has resolved, and I am grateful that the problem was found and treated without needing a bigger operation.”

## Data Availability

The raw data supporting the conclusions of this article will be made available by the authors, without undue reservation.

## References

[1] Aldawas G Ragab S. A review of thyroid rests: from embryology to clinical management. Cureus. (2025) 17(10):e95027. 10.7759/cureus.95027 41127496 PMC12538268

[2] Guerra G Cinelli M Mesolella M Tafuri D Rocca A Amato B. Morphological, diagnostic and surgical features of ectopic thyroid gland: a review of literature. Int J Surg. (2014) 12(Suppl 1):S3–S11. 24887357 10.1016/j.ijsu.2014.05.076

[3] Kajihara K Kawabata T Koga H Marutsuka K Okuda T Ide S. Ectopic thyroid carcinoma presenting as a superior mediastinal cystic mass. Cureus. (2025) 17(10):e94823. 10.7759/cureus.94823 41250727 PMC12620037

[4] Tej V Kummari S Arora AJ Srirambhatla A Ashfaq S. Triple-Site ectopic thyroid involving the submandibular, lingual, and infrahyoid regions: a rare case. Cureus. (2025) 17(10):e94059. 10.7759/cureus.94059 41210011 PMC12591692

[5] Wei C Zhao Y Song Y Liu D Zhang N Liu J. A rare case of an intracardial ectopic thyroid in the right ventricle. J Cardiovasc Dev Dis. (2025) 12(2):45. 10.3390/jcdd12020045 39997479 PMC11855985

[6] Zhao L Zhao L Hu X Wang Z Zhan J. Biliary atresia with ectopic thyroid in the porta hepatis: a case report and literature review. Transl Pediatr. (2024) 13(11):2059–66. 10.21037/tp-24-307 39649640 PMC11621884

[7] Liu Y Chen K Chen M Yang C. Ectopic thyroid tissue in duodenum-A rare case in a young woman. Am J Gastroenterol. (2021) 116(3):448. 10.14309/ajg.0000000000001040 33136572

[8] Mamone G Caruso S Milazzo M Miraglia R. Case 306: ectopic thyroid goiter in the porta hepatis. Radiology. (2022) 305(1):242–6. 10.1148/radiol.210539 36154285

